# Non-Contact Blood Pressure Monitoring Using Radar Signals: A Dual-Stage Deep Learning Network

**DOI:** 10.3390/bioengineering12030252

**Published:** 2025-03-02

**Authors:** Pengfei Wang, Minghao Yang, Xiaoxue Zhang, Jianqi Wang, Cong Wang, Hongbo Jia

**Affiliations:** 1 Air Force Medical Center, Air Force Medical University, Beijing 100036, China; wangpf2016@fmmu.edu.cn (P.W.); minghaoyang_fmmu@163.com (M.Y.); ktzxx1008@163.com (X.Z.); 2 Dujiangyan Special Service Nursing Center of Air Force, Chengdu 611800, China; 3 Department of Military Biomedical Engineering, Air Force Medical University, Xi’an 710032, China; wangjq@fmmu.edu.cn

**Keywords:** non-contact, blood pressure measurement, deep learning, radar, residual networks, transformer

## Abstract

Emerging radar sensing technology is revolutionizing cardiovascular monitoring by eliminating direct skin contact. This approach captures vital signs through electromagnetic wave reflections, enabling contactless blood pressure (BP) tracking while maintaining user comfort and privacy. We present a hierarchical neural framework that synergizes spatial and temporal feature learning for radar-driven, contactless BP monitoring. By employing advanced preprocessing techniques, the system captures subtle chest wall vibrations and their second-order derivatives, feeding dual-channel inputs into a hierarchical neural network. Specifically, Stage 1 deploys convolutional depth-adjustable lightweight residual blocks to extract spatial features from micro-motion characteristics, while Stage 2 employs a transformer architecture to establish correlations between these spatial features and BP periodic dynamic variations. Drawing on the intrinsic link between systolic (SBP) and diastolic (DBP) blood pressures, early estimates from Stage 2 are used to expand the feature set for the second-stage network, boosting its predictive power. Validation achieved clinically acceptable errors (SBP: −1.09 ± 5.15 mmHg, DBP: −0.26 ± 4.35 mmHg). Notably, this high degree of accuracy, combined with the ability to estimate BP at 2 s intervals, closely approximates real-time, beat-to-beat monitoring, representing a pivotal breakthrough in non-contact BP monitoring.

## 1. Introduction

Global epidemiological studies [1] report a twofold escalation in hypertension incidence over three decades (1990–2020), surpassing 1.3 billion cases globally, with underdiagnosis rates exceeding 40. Given an aging global population, hypertension has become an urgent public health concern [2]. As a vital biomarker of circulatory homeostasis, blood pressure (BP) reflects real-time cardiovascular performance and systemic hemodynamic equilibrium. Continuous BP monitoring can enhance early diagnosis of hypertension and related cardiovascular diseases, track treatment efficacy, and ultimately reduce both incidence and mortality rates [1,3].

Conventional cuff-based techniques, while widely adopted, face inherent limitations in capturing transient BP fluctuations due to their intermittent operation mode, thereby necessitating the development of novel solutions for continuous monitoring [4]. Hemodynamic analysis via pulse wave characteristics (e.g., propagation velocity, arterial compliance indices) has gained traction as a non-invasive strategy for continuous BP estimation, leveraging time-domain and morphological features of vascular pulsations [5,6]. Various sensors, including photoplethysmography (PPG) [7], electrocardiogram (ECG) [8], ultrasound [9], and pressure sensors [10,11], have been utilized to capture stable pulse waves for BP estimation. However, these methods often require direct skin contact, which can cause discomfort and limit their use in patients with burns, or contagious diseases like COVID-19.

**Figure 1:**
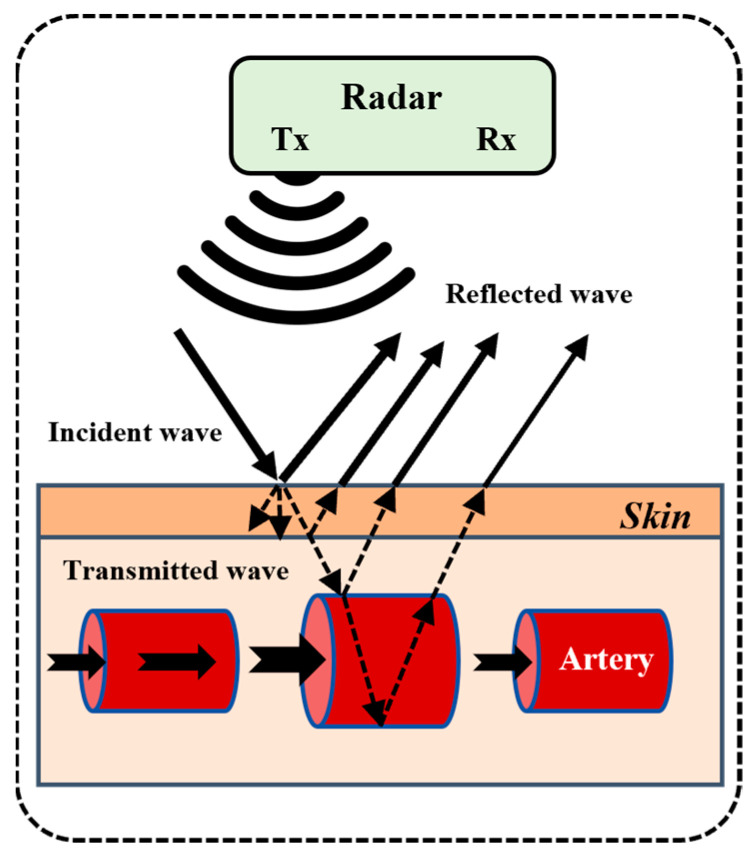
Principle of BP detection based on radar.

This limitation has spurred interest in non-contact approaches, with radar-based BP detection technologies gaining traction among researchers. Radar-based BP measurement employs electromagnetic waves to non-invasively capture cardiovascular dynamics through dual coupled mechanisms (Figure 1): tissue-penetrating detection of cardiac/arterial pulsations [12] and Doppler-based sensing of submillimeter skin vibrations induced by pulse waves. By correlating these dual-modality signals with empirically validated physiological models, the system enables contactless BP estimation. Radar technology enables the contactless acquisition of BP-related biomechanical signals, circumventing the limitations of conventional techniques that require direct skin adhesion or cuff inflation. This capability is particularly advantageous for patients with dermatological conditions or contagious diseases, where direct sensor contact is contraindicated.

Despite its advantages, radar-derived BP signals are prone to degradation from physiological noise (e.g., respiratory modulation, musculoskeletal tremors), making it challenging to extract weak pulse signals and affecting the accuracy and stability of BP measurement models. Previous studies have explored the feasibility of using radar to measure weak pulse waveforms [13,14,15,16] and pulse wave velocity [17,18,19], with the accuracy of the measurements validated against certified devices as a benchmark.

Current radar-based BP modeling strategies fall into two distinct methodological methods:

(1) Biophysical modeling paradigms. Early approaches primarily relied on biophysical equations (e.g., Moens–Korteweg equation) to link pulse wave metrics (e.g., transit time, propagation velocity) with BP values. For instance, hybrid sensor configurations integrating radar with ECG or PPG were explored to derive pulse transit time (PTT) [20,21,22], while standalone radar systems captured pulsatile signals from the chest, radial artery, and multi-site anatomical regions [23,24,25,26,27,28,29]. While these studies employed linear regression to map PTT to BP, their reliance on simplified hemodynamic assumptions has constrained adaptability to inter-subject variability and dynamic cardiovascular states, particularly in accounting for individual arterial compliance differences and transient hemodynamic responses such as exercise-induced BP fluctuations.
(2) Machine learning-driven hemodynamic mapping. Contemporary data-driven paradigms employ deep neural networks to autonomously decode non-linear relationships between radar waveforms and BP dynamics. In contrast, conventional machine learning techniques (e.g., SVM with ensemble methods [30], decision trees [31], artificial neural networks [32,33,34], and random forests [35,36]) necessitated labor-intensive feature engineering, often requiring domain expertise to curate optimal feature sets from time-frequency signal representations. Manual processes can introduce significant user bias and algorithmic sensitivity, affecting BP monitoring outcomes. In contrast, deep learning techniques offer end-to-end BP prediction, bypassing the need for intricate feature engineering. Y. Ran [37] employed a convolutional neural network (CNN) to extract arterial pulse features and built an encoder-decoder neural network for BP estimation, while our preliminary work [38,39] combined CNN with Recurrent neural network networks for pulse feature extraction and BP prediction. However, the accuracy in both cases was not ideal, highlighting the limitations of shallow convolutional networks in feature extraction. Prior attempts to apply ResNet architectures [40] prioritized training on extensive datasets (e.g., 90% of samples), yet overfitting risks emerged in validation scenarios. Wang et al. converted radar signals into images and utilized a U-Net architecture for BP prediction [41]. Similarly, deformable convolutional networks [4] achieved higher accuracy by aggregating long-time-window features but incurred latency penalties, hindering real-time monitoring capabilities. These limitations underscore the need for architectures that balance temporal resolution with computational efficiency.

To address these challenges, we propose a cascaded neural framework that hierarchically extracts spatiotemporal pulse signatures. In the inaugural stage, the radar chest micro-vibration signal (RCMV) and its second derivative (SDRCMV) are directed into a ResNet model, paving the way for foundational estimations of systolic (SBP) and diastolic (DBP) blood pressure values. Then, a transformer model deciphers the intricate pulsatile wave propagation kinetics within the signals, thereby significantly elevating the precision of SBP and DBP determinations.

This study introduces significant advancements in the realm of non-contact BP monitoring through the innovative integration of radar technology with deep learning methodologies. The core contributions can be summarized as follows:

(1) A tailored preprocessing pipeline is developed, incorporating band-pass filtering and trend decomposition to isolate cardiogenic vibrations from respiratory artifacts and motion noise. Initially, the RCMV undergoes selective amplification through band-pass filtering and trend decomposition, separating it from respiratory noise. The refined RCMV, complemented by its SDRCMV, constitutes dual-channel inputs primed for enriched feature extraction within the subsequent deep learning framework.
(2) A deformable ResNet architecture is designed to disentangle multi-resolution spatiotemporal signatures from chest wall micromotions. Leveraging the inherent strength of ResNets in capturing spatial features, this approach facilitates the adaptive extraction of relevant features crucial for BP estimation.
(3) A transformer-enhanced temporal module implements inter-signal coherence analysis, capturing time dependencies across pulse waveform segments and dynamically weighting critical segments of the pulse waveform. By effectively harnessing these capabilities, the model accurately monitors BP changes over time, leading to the construction of more precise and robust BP estimation models.
(4) Capitalizing on the physiological coupling between systolic and diastolic pressures [42], our framework implements a cascaded prediction strategy, where initial estimates of BP obtained from the first stage ResNet are used to augment the feature inputs for the subsequent stage. This strategy enhances the predictive power of the model, resulting in more accurate BP predictions.

This paper is organized as follows: Section 2 reviews preprocessing techniques for radar-based physiological signals and details dual-stage deep neural network architecture. Section 3 quantifies the model’s clinical validity through Bland–Altman analysis and error distribution metrics using clinical-grade reference benchmarks. Section 4 compares the approach to existing methods and quantifies the contribution of different components. Section 5 explores the potential applications of the proposed method.

## 2. Materials and Methods

In this section, we outline the essential data preprocessing steps, signal correlation analysis, and the proposed model.

### 2.1. Data Preprocessing

#### 2.1.1. Dataset Information

In this study, we utilized the physiological signal database from [43]. The dataset, sourced from 30 healthy subjects, includes synchronized radar and BP measurements sampled at 2000 Hz and 200 Hz, respectively. Experimental protocols covered resting states, breath-holding, Valsalva maneuvers, and tilt-table tests across multiple body orientations.

**Figure 2:**
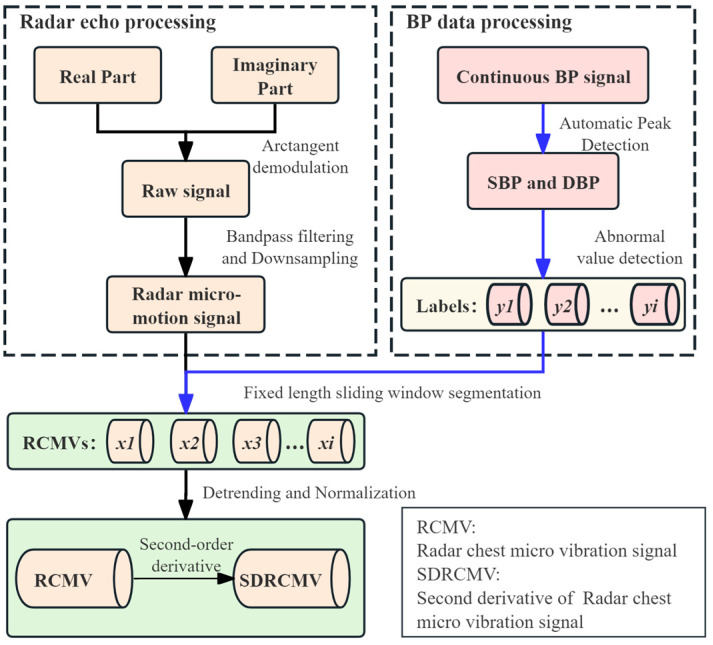
Overview of dataset preprocessing.

Figure 2 shows the preprocessing flowchart of the proposed methodology. Central to this process are the RCMV and reference BP signals, which function as the primary input modalities for the algorithmic framework. The radar signal and reference BP signal implemented fixed-duration windowing (2 s duration) with guard intervals to prevent spectral leakage. Within these individual windows, the radar signal underwent a meticulous preprocessing regimen involving filtering and trend decomposition techniques. This sequence of operations is pivotal in mitigating the effects of low-frequency components, ultimately yielding a purified RCMV signal, providing the foundation for subsequent analytical procedures.

#### 2.1.2. Physiological Signal Preprocessing

The dynamic BP data incorporated within the dataset were acquired through the utilization of continuous non-invasive arterial pressure (CNAP) technology, recognized for its sensitivity. Given the range of experimental conditions subjects were exposed to during data collection, the dataset inevitably incorporates transiently atypical BP readings. Furthermore, a few subsets of the data lack SBP/DBP labels, introducing additional challenges. To mitigate these limitations, we adopted a peak identification protocol [44], with careful consideration of the dynamic morphological characteristics of the BP signal. This method not only allowed us to expand the number of usable data samples, enhancing the overall utility and robustness of the dataset but also facilitated the accurate identification and location of persistent outlier data along with their respective positional indices within the dataset. After synchronizing the timing of radar with dynamic BP signals, those signals that were directly connected to the outlier indices were systematically eliminated, thereby preserving the integrity and dependability of the dataset.

Based on the clinical standards outlined by the American Heart Association and the American Society of Hypertension (SBP: 90–130 mmHg, DBP: 60–90 mmHg), we classified BP measurements exceeding 180 mmHg or dropping below 40 mmHg as outliers within our dataset of healthy participants. Notably, the BP range observed in this cohort extended beyond the thresholds specified in [4], introducing additional complexity. This broader range poses a substantial challenge to the model’s ability to achieve stable feature representation and robust performance while maintaining accuracy and relevance.

#### 2.1.3. Radar Signal Preprocessing

As depicted in Figure 2, the workflow progressed from the initial acquisition of radar signals to the ultimate generation of a functional signal dataset. The radar signal was initially retrieved via arctangent demodulation. To isolate pulse-related components, a bidirectional 4th-order Butterworth band-pass filter (0.5–15 Hz passband) was applied, followed by downsampling to 150 Hz for computational efficiency. The peak within the ambulatory BP signal was then harnessed as a temporal landmark, serving to delineate the starting index for signal segmentation. By employing a fixed-time window approach, the corresponding radar signal was segmented, with an effort to ensure that the segmentation process remained devoid of repetition.

This study employed the characteristic frequency bands of physiological signals to refine radar data, with a principal focus on extracting the RCMV signal. The second derivative of the digital photoplethysmogram (SDPTG), acknowledged as a critical indicator of arterial stiffness [45], demonstrated a strong correlation with fluctuations in BP. Capitalizing on this foundational knowledge, the SDRCMV signal was calculated and integrated as a supplementary input into the proposed algorithm. This enhancement bolstered the algorithm’s feature extraction capabilities, facilitating more nuanced analysis.

A broader frequency range was employed during band-pass filtering, with the intent to preserve the micro-vibration components such as heartbeats, avoiding their erroneous elimination. However, this inclusive approach could inadvertently result in the retention of certain low-frequency DC components within the radar signal post-processing. To counteract this issue and achieve a cleaner signal output, a trend decomposition technique was incorporated following the segmentation of the signal. Denoting *x_i_* as the *i*th observation of the radar time series signal, this can be expressed as follows:

*x_i_* = *α*_0_ + *α*_1_*t_i_* + *ξ_i_*. (1)

where *α*_0_ and *α*_1_ represent the intercept and slope terms, respectively. *ξ_i_* is the error term. The detrended signal is obtained by the following:

*detrend*(*x*) = *x* − (*α*_0_ + *α*_1_ *t*). (2)

Following the meticulous elimination of residual low-frequency components from the radar signal, the second-order derivative was calculated, serving as an enhanced feature for the algorithm’s secondary channel:

(3) $$y^{\prime \prime }[n]=\left(y[n+1]-2y[n]+y[n-1]\right)/{\left(T_{s}\right)}^{2}$$

where *y*[*n*] is sampled at discrete points *n*, and *T_s_* is the sampling period.

To optimize these features for model input, we applied feature scaling through L2-normalization. This streamlined process ensured uniformity and reduced sensitivity to magnitude differences, making it ideal for machine learning models.

### 2.2. Signal Correlation Analysis

#### 2.2.1. Correlation Analysis Between Radar and Blood Pressure Signal

**Figure 3:**
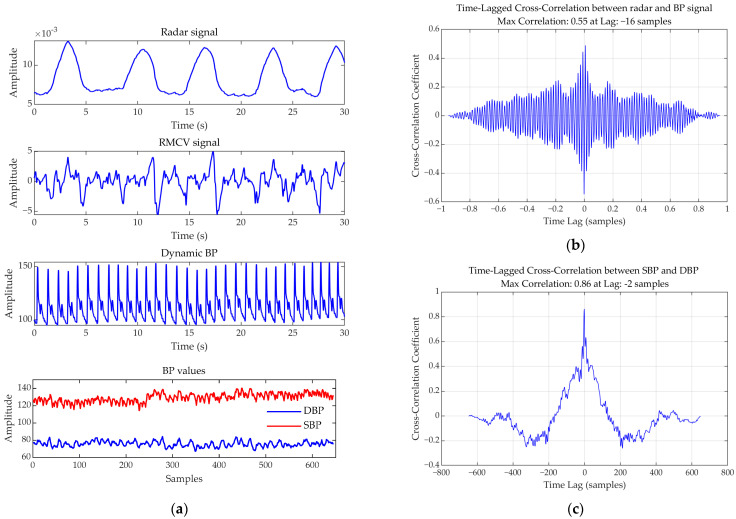
Signal correlation analysis. (**a**) The radar echo signal, the extracted RCMV signal, and the dynamic BP signal from a single subject. (**b**) Time lag cross-correlation analysis between RCMV and dynamic BP signal. (**c**) Time lag cross-correlation analysis between SBP and DBP.

The radar echo signal from a single subject, along with the extracted RCMV signal and the dynamic BP signal, are illustrated in Figure 3a. To investigate the relationship between radar signals and BP, we conducted a Pearson correlation analysis on data from a single subject. We employ linear covariance analysis *ρ_xy_* where *ρ* ∈ [−1, 1] quantifies linear dependence [46].

(4) $$\rho_{xy}=\frac{\sum_{i}\left(x_{i}-\bar{x})(y_{i}-\bar{y}\right)}{\sqrt{\sum_{i}{\left(x_{i}-\bar{x}\right)}^{2}{\left(y_{i}-\bar{y}\right)}^{2}}}$$

Our analysis revealed a correlation coefficient of −0.404 (P = 0) between radar signals and dynamic BP, indicating a moderate negative linear relationship.

To address potential temporal misalignment, a TLCC analysis was conducted [47]. The maximum correlation coefficient (0.55) occurred at an optimal lag (Figure 3b), underscoring the necessity of time-shifted synchronization for accurate BP estimation.

#### 2.2.2. Correlation Analysis Between SBP and DBP

We analyzed the intrinsic relationship between SBP and DBP using Pearson correlation. Equation (4) was employed to calculate the Pearson correlation coefficient between SBP and DBP, yielding a value of 0.6989, indicating a strong positive correlation between these two parameters.

To investigate potential temporal dynamics caused by systematic errors, we conducted a TLCC analysis on SBP and DBP. The results, presented in Figure 3c, show that the maximum correlation coefficient reached 0.86, further confirming the strong intrinsic relationship between SBP and DBP. These findings suggest that leveraging this correlation can enhance the accuracy of BP estimation.

### 2.3. Model Architecture

**Figure 4:**
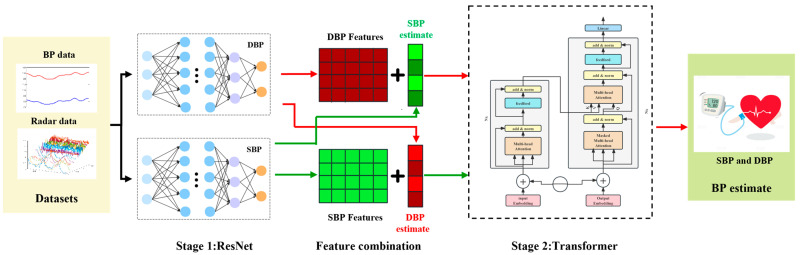
Overview of the structure of a two-stage BP estimation network.

From the perspective of waveform spatial features and time-dependent information extraction, the proposed two-stage BP estimation network is depicted in Figure 4. The architecture encompasses a ResNet module for feature extraction and initial BP prediction, a feature fusion component, and a transformer network dedicated to the extraction of time series information and subsequent BP estimation. The network structure of each part is detailed below.

#### 2.3.1. ResNet Network

**Figure 5:**
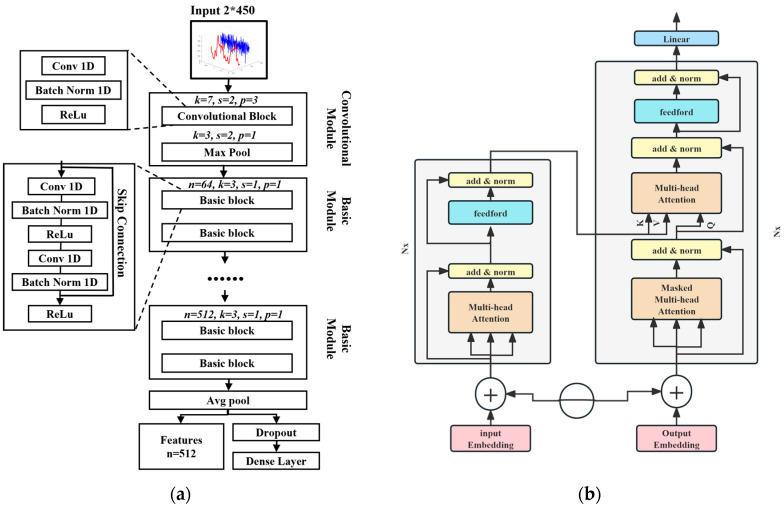
(**a**) Graphical representation of the ResNet model. (**b**) Graphical representation of the transformer model.

Drawing inspiration from the pioneering research of He et al. [48], the proposed ResNet module leverages residual blocks as its core architectural element. This innovative design facilitates enhanced propagation of gradients, which is crucial for training deep neural networks. By employing skip connections, the network is empowered to learn residual functions, which are additive to the input, rather than attempting to fit the entire input-to-output mapping directly. This mechanism is encapsulated in the basic residual block formulation:

(5) $$y=F(x,\left\{W_{i}\right\})+x,$$

where **x** and **y** are the input and output vectors of the layers. Figure 5a shows the two convolutional layers in a basic block. The function F(**x**, {*W_i_*}) represents the residual mapping to be learned.

To mitigate the degradation problem inherent in deep networks, ResNet-18 was selected for its ability to balance computational efficiency with predictive accuracy.

A key advantage of ResNet lies in its adaptability. By modifying hyperparameters like the depth of residual blocks and layer configurations, the network’s width and depth can be tailored to achieve varying levels of representational power, making it adaptable to different datasets. In pursuit of an optimal balance between performance and computational efficiency, this study opted for the ResNet-18 variant to execute the initial phase of BP estimation and feature extraction. Figure 5a provides a comprehensive visualization of the network’s architecture, including layer-specific parameters and structural details.

Following the ResNet network’s processing, eigenvector sets specific to SBP and DBP are derived post-average pooling. Denoted as **F**_S_ = [*F_S_*_1_, *F_S_*_2_, …, *F_Sn_*] for SBP and **F**_D_ = [*F_D_*_1_, *F_D_*_2_, …, *F_Dn_*] for DBP, these vectors encapsulate pertinent features extracted by the network. Following the dense layer, preliminary BP estimations, ***Y***_S_ = [*y_S_*_1_, *y_S_*_2_, …, *y_Sn_*] and ***Y****_D_* = [y_D1_, y_D2_, …, y_Dn_], are generated. These initial predictions are then integrated with the feature vectors to form comprehensive feature matrices. For SBP, **X**_S_ = {*F_S_*_1_, *F_S_*_2_*,* …, *F_Sn_*, *Y_D_*} and **X**_D_ = {*F_D_*_1_*, F_D_*_2_, …, *F_Dn_*, *Y_S_*} are feature matrices that serve as the input dataset for the second stage of the BP sample, embodying a strategic fusion of spatial features and initial BP estimates to enhance subsequent temporal analysis and final BP estimation accuracy.

#### 2.3.2. Transformer Architecture

Following Vaswani et al.’s seminal work [49], the transformer architecture is integrated into the second stage, harnessing context-aware attention mechanisms to decode temporal dependencies among all input elements. Figure 5b illustrates the transformer architecture applied and adapted for the BP monitoring task.

The transformer architecture comprises several key components: an input embedding layer, positional encoding, an encoder featuring multi-head self-attention (MHA) and a feed-forward network, a decoder consisting of masked MHA and cross-modal attention gates between physiological features and temporal contexts, and an output layer.

An embedding layer first transforms the input features via linear projection, standardizing their dimensionality for subsequent processing. Mathematically, this operation can be expressed as follows:

*E*(**X**) = **XW**_embed_ +*b*_embed_, (6)

where *E* is the embedded representation, **X** is the input feature matrix, **W**_embed_ is the weight matrix for the embedding layer, and *b*_embed_ is the bias term. This linear projection enables the network to interpret and manipulate the input features in a way that maximizes the extraction of meaningful temporal relationships.

While traditional recurrent models inherently capture sequence order, transformers require explicit positional encoding to inject temporal information into the input embeddings. The positional encoding is employed to imbue the input sequences with information regarding the relative positions of tokens. This is particularly critical in the context of physiological signal analysis, where the temporal dependencies among data points hold significant value. The sine-cosine positional encoding scheme is adopted, and the mathematical formulation is given by the following:

(7) $$\begin{array}{l} PE_{\left(pos,2i\right)}=sin(pos/10000^{2i/d_{model}})\\ PE_{\left(pos,2i+1\right)}=cos(pos/10000^{2i/d_{model}}) \end{array},$$

where *pos* is the position and *i* is the current feature dimension of the input data. Model refers to the total number of dimensions in the feature space. The encoding strategy ensures that the transformer model remains sensitive to the temporal dynamics of the input physiological signals, a crucial factor in achieving high-fidelity BP estimation.

To effectively capture the intricate relationships within the input sequence and distill contextual information at each position, the self-attention mechanism is applied. This mechanism, a cornerstone of the transformer architecture, is pivotal in understanding the nuanced dependencies within sequences of physiological signals.

Central to the self-attention mechanism is the scaled dot-product attention, complemented by the MHA strategy. This approach enhances the model’s capacity to attend to information at different representation subspaces at different positions. By incorporating positional encoding into the input embeddings, the model gains awareness of the sequence’s temporal structure.

The self-attention mechanism operates on three core components: query (**Q**), key (**K**), and value (**V**), which jointly determine the output through scaled dot-product interactions. The output is computed as follows:

(8) $$Attention(Q,K,V)=softmax(\frac{QK^{T}}{\sqrt{d_{k}}})V,$$

where *d*_k_ is the dimension of **K**.

Let **W_i_^Q^** ∈ $R^{d_{\mathrm{model}}\times d_{k}}$, **W_i_^K^** ∈ $R^{d_{\mathrm{model}}\times d_{k}}$, **W_i_^V^** ∈ $R^{d_{\mathrm{model}}\times d_{v}}$ be the respective learned weights for the three attention vectors **Q**, **K**, and **V**, respectively, corresponding to the *i*th head. **W**_O_ ∈ $R^{hd_{v}\times d_{\mathrm{model}}}$ is the weight for the output MHA. Then, the *i*th head may be defined as follows:

(9) $$head_{i}=Attention(QW_{i}^{Q},KW_{i}^{K},VW_{i}^{V}).,$$

Attention from different subspaces (heads) [49] is subsequently concatenated to achieve the final *MHA* output:

(10) $$Multihead(Q,K,V)=Concat(head_{1},\ldots ,head_{h})W^{O},$$

Analogous to the *MHA* mechanism, the *MHA* module within the decoder is defined as follows:

*MHA*(**Q**, **K**, **V**) = concat(**A**_1_, **A**_2_,…, **A***_H_*)**W^O^**, (11)

where **A**_h_ = attention(**Q**_h_, **K**_h_, **V**_h_) is the output for the head. The outputs of all headers are connected and linearly projected using the learned weight matrix $W_{h}^{O}\in R^{Hd_{v}\times d_{\mathrm{model}}}$ to form the final output of the *MHA* module.

Each layer within both the encoder and decoder components of the network incorporates a fully connected feed-forward network (*FFN*). This network is applied independently to each position within the sequence, allowing for parallel computation while maintaining the same parameters across all positions. The *FFN* is comprised of two linear transformations separated by a rectified linear unit (ReLU) activation function, defined as follows:

*FFN*(*x*) = max(0, *x***W**_1_ + *b*_1_)**W**_2_ + *b*_2_, (12)

where **W**_1_ and *b*_1_ are the weights and offsets of the first full connection layer, and **W**_2_ and *b*_2_ are the weights and offsets of the second full connection layer. The incorporation of FFNs serves to augment the non-linear capabilities of feature representation, enabling the model to learn intricate mappings between the input and output spaces. This enhancement is critical for accurately modeling the subtle dynamics present in physiological signals, thereby contributing to the overall effectiveness of the BP monitoring system.

Layer normalization independently normalizes the feature values of each sample within a layer. This operation is mathematically formalized as follows:

(13) $$\mathrm{LayerNorm}=\gamma (\frac{x-\mu }{\sigma })+\beta ,$$

where *μ* and *σ* are the mean and variance of eigenvalues, and *γ* and *β* are learnable parameters. The application of layer normalization in both the encoder and decoder sections accelerates the training process significantly. Stabilizing the distribution of activations across layers mitigates issues such as rapid changes in gradients, thereby enhancing the model’s convergence speed and overall stability.

## 3. Experiments and Results

### 3.1. Dataset

The dataset encompassed a cohort of 30 volunteers (16 female, 14 male). The age of the subjects ranged from 21 to 61 years old, with a mean age of 30.7 years old; height ranged from 161 cm to 193 cm, with a mean height of 175.7 cm; weight ranged from 44 kg to 94 kg, with a mean weight of 72.2 kg; and BMI ranged from 18.6 to 31.4, with a mean BMI of 23.2. All physiological recordings were collected at the University Hospital of Erlangen under a standardized protocol. Subjects underwent various physiological states, including rest, the Valsalva maneuver, apnea, and the tilt-table test, to elicit a range of cardiovascular responses [43].

**Figure 6:**
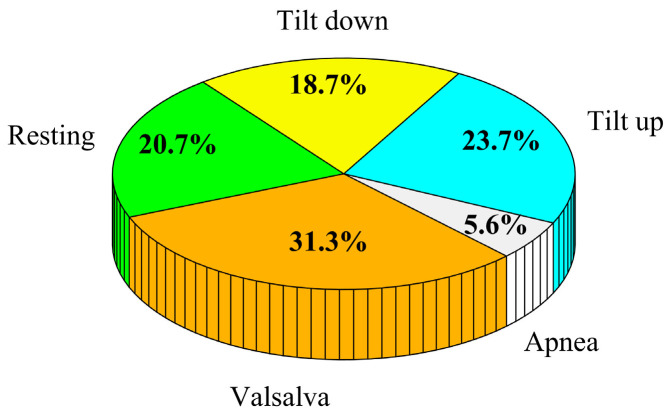
Schematic diagram of the data distribution ratio in the dataset under different action states.

After the data preprocessing process, the sample distribution in different data collection scenarios is shown in Figure 6. Following this segmentation process, we acquired more than 40,000 samples, ensuring an ample dataset size that is well-suited for the effective training of deep neural networks. According to the conventional practices in machine learning for dataset partitioning, the dataset was partitioned into three subsets: 65% for training, 15% for validation, and 20% for testing, ensuring no overlap between groups.

### 3.2. Training

Model inputs comprised two signal modalities: RCMV and SDRCMV. The training spanned 60 epochs via PyTorchon 3.8.20 dual NVIDIA 3080Ti GPUs (Nvidia, CA, USA). During training, the test set was isolated to ensure an unbiased evaluation of the model’s performance. The initial learning rate was set to 0.001, with adaptive reduction triggered by the Plateau scheduler (factor = 0.5) if validation loss plateaued for >5 epochs. Adam optimizer was employed with decoupled weight decay, and an L2 regularization coefficient of 0.002 was applied to prevent overfitting. Early stopping based on validation loss was also enabled to further enhance generalization. To optimize resource utilization, batch sizes were configured as 32 (Stage 1) and 4 (Stage 2), balancing speed and accuracy. These settings ensured stable convergence and optimal performance while maintaining practicality and reproducibility across different platforms.

### 3.3. Criterion

To optimize model training, we adopted the Pseudo Huber loss function [50], an adaptive robust loss with continuous curvature that combines robustness against outliers with stable gradient properties:

(14) $$Loss(a,\delta )=\delta^{2}(\sqrt{1+{\left(\frac{a}{\delta }\right)}^{2}}-1),$$

where *a* is the error between the predicted value and the true value, and *δ* is a hyperparameter used to control the balance between robustness and sensitivity to data changes. It combines the advantages of the Huber loss function and the mean square error (MSE) loss function to provide a smooth transition between the quadratic loss of small errors and the linear loss of large errors. This loss function minimizes the impact of anomalous data points by attenuating their contribution to the gradient updates. This safeguard promoted better generalization to unseen data, minimizing overfitting risks. Thus, model training was more stable and dependable.

### 3.4. Performance Evaluation of the Model

Model accuracy was quantified via mean error (*ME*), mean absolute error (*MAE*), and root mean square error (*RMSE*), aligned with (BHS) guidelines for BP measurement validation:

(15) $$ME=\frac{1}{n}\sum_{i=1}^{n}(y_{i}-{\hat{y}}_{i}),$$

(16) $$MAE=\frac{1}{n}\sum_{i=1}^{n}\left|y_{i}-{\hat{y}}_{i}\right|,$$

(17) $$RMSE=\sqrt{\frac{1}{n}\sum_{i=1}^{n}{\left(y_{i}-{\hat{y}}_{i}\right)}^{2}},$$

where *y_i_* represents the true value, ${\hat{y}}_{i}$ represents the predicted value, and *n* is the total number of test samples.

Table 1 summarizes model performance across varying input window durations (t = 2 s, 3 s, 4 s). Upon testing, the models demonstrated notably high accuracy rates, peaking when t = 2 s.

Following the BHS criteria, which evaluates accuracy through the cumulative frequency of errors, a systematic analysis was performed on data with varying input durations. As presented in Table 2, the model achieved an “A” grade at t = 2 s. For SBP, the error frequencies were 62.74%, 87.42%, and 95.37% (<5 mmHg, <10 mmHg, <15 mmHg), while for DBP, they were 75.49%, 93.15%, and 97.35%. Both exceeded the “A” grade thresholds of 60%, 85%, and 95%, confirming the model’s superior performance. Notably, when t = 3 s and t = 4 s, the model closely approached the “A” rating, consistently meeting the “B” standard. This is indicative of high accuracy despite the slight increase in error margin.

According to the AAMI standard, BP devices must achieve a ME within ±5 mmHg and a standard deviation (SD) below 8 mmHg for both SBP and DBP measurements. Our method demonstrates compliance with these requirements, achieving ME values of −1.09 mmHg (SBP) and −0.26 mmHg (DBP), along with SD values of 5.15 mmHg (SBP) and 4.35 mmHg (DBP), thereby validating its clinical-grade accuracy.

To prevent overfitting and enhance generalization, we implemented several strategies in our experimental setup. An adaptive learning rate reduction (factor = 0.5) was triggered by the Plateau scheduler if validation loss plateaued for more than 5 epochs. We used the Adam optimizer with decoupled weight decay and applied L2 regularization with a coefficient of 0.002 to constrain model complexity. Additionally, early stopping based on validation loss ensured training terminated when no significant improvement occurred. These methods collectively improved the model’s robustness and generalization capability.

Extended time windows reduced usable samples, likely contributing to diminished precision at t = 3 s and t = 4 s. This phenomenon suggests that larger time frames, while potentially capturing more comprehensive data, could inadvertently limit the volume of analyzable samples, thus impacting the precision of the results.

**Figure 7:**
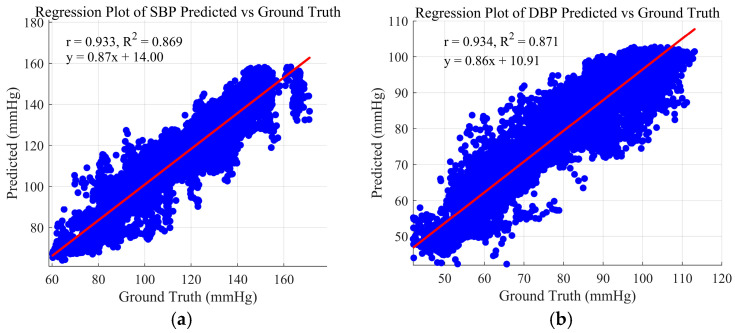
Regression plot of predicted and actual values for SBP (**a**) and DBP (**b**).

Figure 7 illustrates the linear concordance between predicted and reference measurements, with dispersion plots with Pearson’s (*r*) and coefficient of determination (R^2^) for SBP (a) and DBP (b). Each data point in the regression scatter plot corresponds to a comparison between the predicted and actual values. Specifically, a high r value indicates a strong positive correlation. Furthermore, Figure 7 provides the best-fit linear equation for the relationship between the predicted and true values, with the slope and intercept of the regression line quantitatively defining this association.

**Figure 8:**
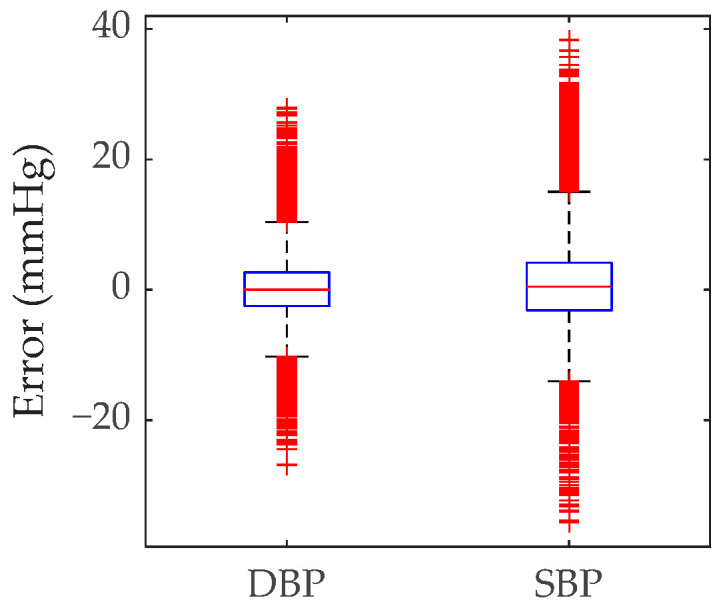
Box plot analysis of prediction errors for SBP and DBP.

Figure 8 quantifies prediction deviations using box plots. The box plots depict the dispersion of these errors. The median errors were clustered around zero, indicating that most predicted values closely align with the actual measurements. However, due to the short window length chosen, a high number of sample signals were tested. Some anomalous samples (indicated by red lines) can be observed in Figure 8 that are out of the range of the box plots.

**Figure 9:**
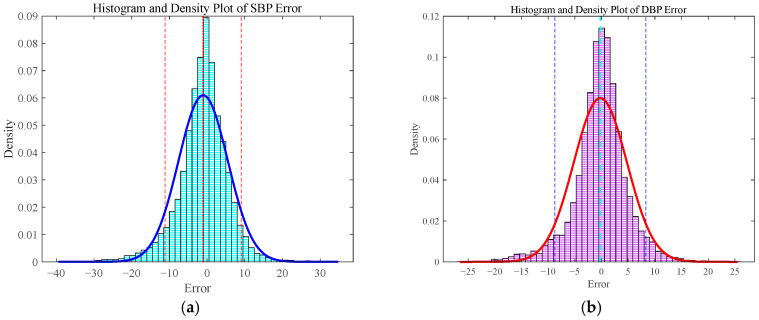
Systematic error distribution analysis for predictions of (**a**) SBP and (**b**) DBP.

Figure 9 presents the histograms and probability density plots illustrating the distribution of prediction errors for both SBP and DBP. The horizontal axis denotes the error, whereas the vertical axis represents the probability density. Histogram analysis reveals a prominent concentration of errors around zero, demonstrating that the majority of predicted values closely match the actual measurements. Furthermore, as previously mentioned, although there were instances where the discrepancy between predicted and actual values was substantial, the probability of encountering errors exceeding ±10 mmHg remained relatively low.

**Figure 10:**
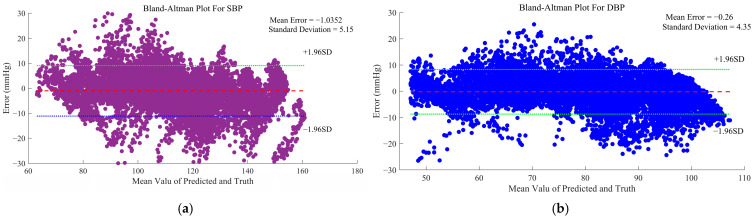
Bland–Altman plots visualizing prediction errors for (**a**) SBP and (**b**) DBP.

Bland–Altman analysis (Figure 10) was performed to assess the agreement between predicted and actual BP values, with the limits of agreement (LOA) calculated as the mean difference ± 1.96 SD depicted in red. The *y*-axis represents the differences between the two measurements, while the *x*-axis displays their averages. Each scatter point on the plot signifies the discrepancy between a sample’s predicted and actual BP values. The plot reveals that while a minority of samples demonstrated substantial discrepancies, the majority of predictions were closely aligned with actual values, with paired measurements falling within the LOA boundaries. Owing to the utilization of the Pseudo Huber loss function, offering a smooth transition between the Huber loss function and the MSE loss function, the gap between robustness and precision has been bridged, achieving significant stability. Notably, the results do not exhibit pronounced consistent bias observed in estimations [4].

### 3.5. Individual Blood Pressure Tracking Analysis Across Varied Motion States

The evaluation of BP across multimodal physiological challenges holds significant value for practical applications. To ascertain the feasibility of the proposed methodology within authentic settings, a detailed examination of single-subject tracking efficacy was conducted. The investigation encompassed a range of subject activities. Utilizing 2 s intervals for near-continuous tracking (≈30 readings/minute), mimicking pulse wave resolution. This temporal precision facilitates the accurate monitoring and quantification of BP dynamics.

**Figure 11:**
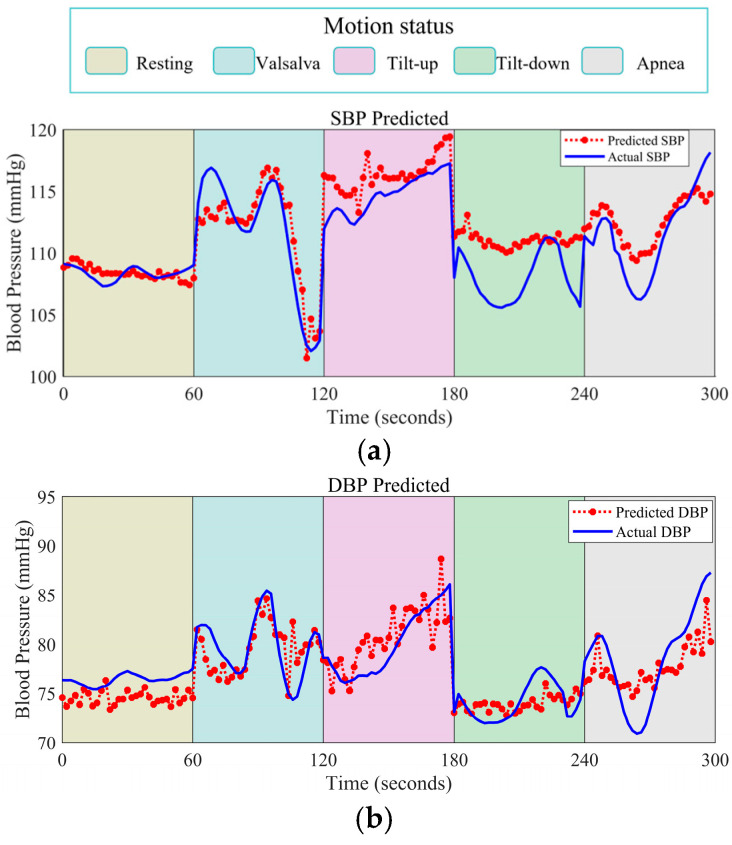
Variation of (**a**) SBP and (**b**) DBP in individual subjects across different motion states tracked using the proposed approach.

As depicted in Figure 11, the novel two-stage BP prediction framework not only captures the overarching trajectory of BP alterations in individual cases, but also exhibits sensitivity to nuanced changes. While acknowledging that certain states yield less precise outcomes, these preliminary findings are nonetheless promising. They underscore the potential of radar-based, non-contact BP detection systems to enable continuous, beat-by-beat BP monitoring, a significant advancement in the field of health monitoring.

## 4. Discussion

To comprehensively appraise the model’s efficacy, the findings of this study were compared against those of existing research, assessing the determinants of model performance. Furthermore, ablation studies were undertaken to assess the individual contributions of each algorithmic component.

### 4.1. Comparative Analysis of Model Performance with Existing Research Benchmarks

This section compares models for non-contact BP measurement, noting considerable dataset disparities linked to methodological heterogeneity arising from distinct sensing principles (UWB/CW), measurement locations (precordial/carotid), and operational bandwidths [4,27,32,33,35,38]. Disparities in data acquisition systems influence crucial aspects, including accuracy and noise. Limited cohorts could inflate accuracy measures via constrained cardiovascular parameter ranges. Hence, this comparative analysis establishes a rudimentary performance reference for modern non-contact BP measurement techniques.

The overall results of this study and the comparison to state-of-the-art methods are shown in Table 3. This contrasts the diverse model performances using critical metrics (ME, MAE, RMSE, STD, and MRE) across various spanning methods, years, positions, and subject counts. ME indicates the mean discrepancies, MAE shows absolute differences that are less outlier-sensitive, RMSE heavily penalizes major deviations through squared errors, and MRE shows relative errors as true value percentages.

Most previous studies have focused on pulse detection and BP prediction during resting scenarios, which simplifies the measurement process and improves accuracy due to minimal BP fluctuations. However, this approach imposes additional constraints on subjects by limiting their movements. Our preliminary research [38] explored various activity scenarios and found that while a convolutional-recurrent hybrid architecture performed well in resting conditions, it struggled in scenarios with significant BP variations. This performance discrepancy is attributed to the limitations of shallow networks in extracting robust pulse features under dynamic conditions.

Although we used the same dataset as [4], our method significantly increased the number of usable data samples, ensuring adequate input for deep learning algorithms by employing a peak detection algorithm during preprocessing and utilizing short-time window segmentation. The results show that, compared to the state-of-the-art method in [4], the MAE for SBP decreased by 10.4%, RMSE by 9.4%, and SD by 1.15 mmHg. Additionally, the MAE and RMSE for SBP measurements achieved near parity (<0.15 mmHg difference), with a further SD improvement of 0.99 mmHg.

Methodologically, our approach leveraged short-window signal segmentation to dynamically monitor continuous blood pressure (BP) variations via radar waveform analysis. By explicitly incorporating physiological continuity priors between adjacent measurements as regularization constraints, we enhanced the model’s sensitivity to transient BP fluctuations. In contrast to [4], which employed fixed long-window processing with 15 s sampling intervals and overlooked hemodynamic temporal correlations, this study achieved a 2 s temporal resolution through short-window segmentation, enabling beat-to-beat measurement and enhancing the ability to detect transient BP changes effectively.

### 4.2. Ablation Studies

#### 4.2.1. ResNet

**Figure 12:**
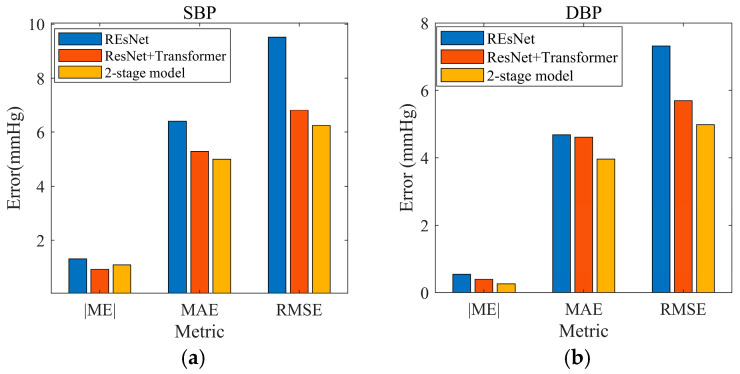
Evolution of predicted error of SBP (**a**) and DBP (**b**): Standard ResNet, ResNet+ transformer, and two-stage model.

Designed as a spatial feature extractor, the modular convolution units have scalable depth/width configurations for radar data adaptation. The pioneering use of skip connections facilitates gradient flow in deep networks, playing a crucial role in extracting detailed features from radar signals. Enhanced with batch normalization, ResNet exhibits commendable resistance to overfitting. However, the continuous temporal dynamics of BP demand additional temporal analysis beyond spatial prowess for accurate estimation. As shown in Figure 12, the RMSE of SBP reached 9.48 mmHg when using the Stage1 network alone, exceeding clinically acceptable tolerance thresholds.

Furthermore, we compared the performance of using the RCMV signal and its first-, second-, and third-order derivatives as inputs, with results summarized in Table 4. We observed that the second derivative, as a supplementary input in the second channel, not only enhances model performance but also provides more effective information compared to the first and third derivatives. This can be attributed to the feature extraction network’s ability to capture features from the RCMV signal that are similar to those of its first derivative, reducing the significance of the first derivative’s contribution when used as the second channel input.

#### 4.2.2. Transformer

The transformer’s attention-based architecture effectively models extended temporal inter-dependencies in time series data, such as radar signals that vary with BP. By computing attention scores across all temporal indices through query-key interactions, the model can consider global information beyond the immediate context, addressing the limitations of RNNs, particularly their susceptibility to gradient instability when handling lengthy sequences.

Positional encoding is a critical component that enables the transformer to understand sequence order, which is essential for interpreting temporal patterns. Meanwhile, the MHA mechanism enhances the model’s representational capacity by enabling parallel self-attention across multiple subspaces. This approach allows the model to focus on both fine-grained details and broader trends, making it highly effective for complex time series analysis.

Integrating the transformer after ResNet feature extraction leverages the temporal richness of BP dynamics, thereby enhancing prediction precision. This strategic use of the transformer’s strengths in sequence processing optimizes the exploitation of temporal cues, leading to superior predictive performance.

#### 4.2.3. Feature Enhancement Using SBP and DBP Correlations

Intrinsically, SBP and DBP in individuals are correlated [42]. By leveraging the physiological coupling between SBP/DBP phases, the Stage-1 network drives feature augmentation through its predictions, thereby enriching the feature set. This integrative approach supplies the model with enhanced discriminative information, establishing the foundation for achieving more precise and reliable BP predictions.

Furthermore, the transformer’s prowess in grasping global and contextual temporal information was harnessed to distill meaningful patterns from the SBP-DBP correlation. This dual-strategy amplified the model’s accuracy in forecasting both SBP and DBP values, showcasing a robust approach to BP prediction.

### 4.3. Limitations and Future Work

Advancing non-contact health monitoring, radar technology is poised to revolutionize BP assessment, operating without patient awareness. This approach mitigates discomfort and bypasses the “white coat effect”, ensuring more accurate readings by eliminating stress-induced elevations typical in clinical environments [51]. A key strength of radar lies in its immunity to external factors like light and temperature, making it ideal for uninterrupted nighttime monitoring [52,53,54]. Particularly noteworthy is its applicability to special populations, including burn victims and those with sensitive skin, offering an alternative to conventional methods.

A key limitation of our research stems from the constrained dataset, which hindered our proposed model’s ability to capture a more extensive spectrum of blood pressure (BP) readings. Although participants reported being in good health, the hemodynamic responses elicited during various experimental motion scenarios covered a BP range of 40–180 mmHg. This range reflects the foundational BP distribution typically encountered in hypertensive individuals, as defined by hypertension practice guidelines [55]. However, this study fell short of including a substantially broader BP measurement range, thus restricting the model’s applicability. Moreover, the dataset omitted pediatric populations (<18 years) and confirmed hypertensive cohorts, leaving questions regarding the model’s effectiveness for these specific groups unanswered. Further validation is therefore required to address these limitations and enhance the model’s generalizability.

Additionally, certain medical conditions, such as isolated systolic hypertension or aortic stenosis, may reduce the correlation between SBP and DBP. Although the transformer mechanism dynamically adjusts the weighting of features related to the correlation between SBP and DBP, the impact of such variations on model performance remains an area requiring further analysis and investigation.

Furthermore, radar-based BP detection grapples with a significant hurdle: closing the accuracy gap with gold-standard sphygmomanometry. Addressing the current issues, we posit that future efforts should focus on the following two aspects:

Firstly, it is essential to establish a population-representative database that includes individuals with diverse health conditions and from various age groups. The correlation between BP and parameters such as PWV, PTT, and pulse waveforms can be significantly modulated by age, gender, body morphology, physical exertion, emotional state, and posture [56,57]. Given that models like transformers are highly data-dependent, compiling a comprehensive database encompassing diverse demographics—including age, gender, BMI, and health status—will be pivotal. Integrating next-generation neural architectures (e.g., graph networks) to construct precise and robust models from this data will pave the way for the practical implementation of radar BP detection.

Secondly, enhancing the resolution of radar systems is crucial for capturing subtle pulse signals. By employing MIMO radar architectures with adaptive beamforming, simultaneous monitoring of pulse information at key arterial sites can provide richer waveform features and more accurate PTT data. This enhancement is expected to significantly improve the precision of BP measurements, bringing radar technology closer to becoming a viable, non-contact standard in healthcare.

## 5. Conclusions

This study has presented a novel dual-stage deep learning architecture for contactless, radar-based continuous BP monitoring. Employing a specialized preprocessing method for radar physiological signals, chest wall vibrations, and their second derivatives were captured, with the dual-channel inputs fed into a hierarchical neural network structure. The framework initially used residual convolutional blocks for localized pattern mining, then progressed to a transformer architecture for sequential dependency modeling, leading to accurate BP estimation.

A key innovation involved integrating ResNet-derived initial BP estimates as augmented feature inputs for downstream network stages, achieving an accuracy improvement through this cross-phase feature fusion approach. Comprehensive validations confirmed the model’s reliability and precision, evidenced by ME values of −1.09 ± 5.15 and −0.26 ± 4.35 mmHg for SBP and DBP, respectively. The proposed model either surpassed or aligned with state-of-the-art methodologies across various metrics, including MAE, RMSE, MRE, and R. The model’s performance met all Level−1 standards according to the BHS protocol.

Notably, the model’s capacity to estimate BP using a 0.5 Hz sampling rate (2 s window) for quasi-continuous tracking approximates real-time, beat-to-beat monitoring, representing a significant advancement in non-contact BP monitoring technology.

Future research will focus on enhancing the model’s adaptability across diverse conditions and patient characteristics while investigating the integration of additional physiological parameters to expand the system’s monitoring capabilities.

## Figures and Tables

**Table 1:** Performance of proposed method with time window lengths t = 2 s/3 s/4 s.

Time window lengths t = 2 s					
SBP			DBP		
ME	MAE	RMSE	ME	MAE	RMSE
−1.09	5.00	6.24	−0.26	3.96	4.98
Time window lengths t = 3 s					
SBP			DBP		
ME	MAE	RMSE	ME	MAE	RMSE
−0.93	5.29	6.87	−0.58	4.06	5.18
Time window lengths t = 4 s					
SBP			DBP		
ME	MAE	RMSE	ME	MAE	RMSE
−0.71	6.68	7.89	−0.39	4.61	5.69

**Table 2:** Performance of the proposed method within the framework of BHS standards.

Cumulative Frequency of Error (%)	<5 mmHg	<10 mmHg	<15 mmHg
Grade A	60	85	95
Grade B	50	75	90
Grade C	40	65	85
SBP (t = 2 s)	**62.74**	**87.42**	**95.37**
DBP (t = 2 s)	**75.49**	**93.15**	**97.35**
SBP (t = 3 s)	**62.75**	**85.92**	94.36
DBP (t = 3 s)	**72.31**	**92.82**	**96.78**
SBP (t = 4 s)	51.20	77.58	88.93
DBP (t = 4 s)	**65.78**	**89.62**	**96.07**

**Table 3:** Statistical comparative evaluation of model performance.

Year	Method	Position	Subject	SBP (mmHg)	DBP (mmHg)	Year	Method	Position	Subject	SBP (mmHg)	DBP (mmHg)	Year	Method
				ME	MAE	RMSE	SD	MRE (%)	ME	MAE	RMSE	SD	MRE (%)
2022	NN [32]	wrist, chest	27	9.51	-	-	12.1	-	-	6.69	-	8.12	-
2022	RF [35]	chest	28	2.52	7.2	-	6.73	-	0.22	6.3		3.85	-
2023	NN [33]	chest	55	9.2	-	11.0	8.3	-	7.7	-	11.0	5.7	-
2023	LS [27]	neck, chest	27	-	5.54	-	7.76	-	-	4.68	-	6.15	-
2024	CNN + LSTM [38]	chest	30	2.04	9.30	-	12.65	-	0.48	5.91	-	8.16	-
2024	SDCN [4]	chest	30	−0.32	4.86	6.14	6.14	4.40	−0.2	4.42	5.5	5.5	5.69
**2025**	**Ours**	**chest**	**30**	**−1.09**	**5.00**	**6.24**	**5.15**	**4.57**	**−0.26**	**3.96**	**4.98**	**4.35**	**5.03**

random forest: RF; neural network: NN; least squares: LS; stacked deformable convolution network: SDCN; mean relative error: MRE.

**Table 4:** Performance of ResNet with different inputs.

Input	SBP			DBP		
	ME	MAE	RMSE	ME	MAE	RMSE
RCMV	−1.08	6.73	9.82	−0.20	5.19	7.41
RCMV with its first derivative	−0.90	6.59	9.71	−0.33	5.08	7.29
RCMV with its second derivative	**−0.88**	**6.40**	**9.48**	**−0.43**	**4.70**	**7.24**
RCMV with its third derivative	−0.95	6.88	9.83	−0.56	5.21	7.43

## Data Availability

Data supporting reported results can be found online (https://figshare.com/articles/dataset/A_dataset_of_clinically_recorded_radar_vital_signs_with_synchronised_reference_sensor_signals/12186516, accessed on 10 December 2023).
